# Biased Agonism of Three Different Cannabinoid Receptor Agonists in Mouse Brain Cortex

**DOI:** 10.3389/fphar.2016.00415

**Published:** 2016-11-04

**Authors:** Rebeca Diez-Alarcia, Inés Ibarra-Lecue, Ángela P. Lopez-Cardona, Javier Meana, Alfonso Gutierrez-Adán, Luis F. Callado, Ekaitz Agirregoitia, Leyre Urigüen

**Affiliations:** ^1^Department of Pharmacology, University of the Basque Country UPV/EHULeioa, Spain; ^2^Centro de Investigación Biomédica en Red de Salud MentalMadrid, Spain; ^3^Department of Animal Reproduction, Instituto Nacional de Tecnología Agraria y AlimentariaMadrid, Spain; ^4^G.I. Biogénesis, Universidad de AntioquiaAntioquia, Colombia; ^5^Department of Physiology, University of the Basque CountryLeioa, Spain

**Keywords:** CB_1_ receptor, CB_2_ receptor, functional selectivity, scintillation proximity assay (SPA), G proteins, brain

## Abstract

Cannabinoid receptors are able to couple to different families of G proteins when activated by an agonist drug. It has been suggested that different intracellular responses may be activated depending on the ligand. The goal of the present study was to characterize the pattern of G protein subunit stimulation triggered by three different cannabinoid ligands, Δ^9^-THC, WIN55212-2, and ACEA in mouse brain cortex. Stimulation of the [^35^S]GTPγS binding coupled to specific immunoprecipitation with antibodies against different subtypes of G proteins (Gα_i1_, Gα_i2_, Gα_i3_, Gα_o_, Gα_z_, Gα_s_, Gα_q/11_, and Gα_12/13_), in the presence of Δ^9^-THC, WIN55212-2 and ACEA (submaximal concentration 10 μM) was determined by scintillation proximity assay (SPA) technique in mouse cortex of wild type, CB_1_ knock-out, CB_2_ knock-out and CB_1_/CB_2_ double knock-out mice. Results show that, in mouse brain cortex, cannabinoid agonists are able to significantly stimulate not only the classical inhibitory Gα_i/o_ subunits but also other G subunits like Gα_z_, Gα_q/11_, and Gα_12/13_. Moreover, the specific pattern of G protein subunit activation is different depending on the ligand. In conclusion, our results demonstrate that, in mice brain native tissue, different exogenous cannabinoid ligands are able to selectively activate different inhibitory and non-inhibitory Gα protein subtypes, through the activation of CB_1_ and/or CB_2_ receptors. Results of the present study may help to understand the specific molecular pathways involved in the pharmacological effects of cannabinoid-derived drugs.

## Introduction

During the last decade a wide number of studies have focused on the potential involvement of the endocannabinoid system in a variety of psychiatric and neurological disorders. The putative psychoactive ingredient of *Cannabis sativa* (marijuana plant), Δ^9^-tetrahydrocannabinol (Δ^9^-THC), as well as the endogenous cannabinoids anandamide (arachidonoyl ethanolamide) and 2-arachidonoylglycerol (2-AG) act primarily through cannabinoid CB_1_ and CB_2_ receptors. These cannabinoid receptors are GPCRs mostly coupled to Gi/o proteins (Howlett et al., 2002). The CB_1_ receptor is mainly distributed in the CNS, particularly in cortex, basal ganglia, hippocampus, and cerebellum (Mackie, 2005; De Jesus et al., 2006) and generally acts presinaptically inhibiting the release of neurotransmitters. CB_2_ receptors are expressed at much lower levels in the CNS compared with CB_1_ receptors (reviewed in Atwood and Mackie, 2010). As G_i/o_ coupled GPCRs, CB_1_ and CB_2_ receptors inhibit adenylyl cyclase, but moreover, both receptors are able to activate MAPK, inhibit voltage gated Ca^2+^ channels and activate inwardly rectifying K^+^ channels (Childers et al., 1993).

The activation of CB_1_ receptor in the brain leads to the modulation of neuronal excitability, which may be in part responsible of the psychoactive effects of exogenous cannabinoids. In this context, a considerable amount of studies have been performed in order to elucidate the effects of cannabinoids (natural or synthetics) in the development of mental alterations, such as addiction, cognitive deficits, anxiety or psychosis. Importantly, different or opposite behavioral effects have been observed after the administration of Δ^9^-THC or synthetic cannabinoid ligands (Fattore et al., 2003; Panagis et al., 2014; Rubino and Parolaro, 2016). It has been demonstrated that for most G protein-coupled receptors, distinct agonists can differentially regulate several signaling pathways through the same receptor by a selective activation of different intracellular effectors. This is a mechanism known as functional selectivity or biased agonism. In this way, cannabinoid receptors have been demonstrated to be capable of coupling to different families of G proteins and/or to beta-arrestin when activated by an agonist drug suggesting that different intracellular responses may be activated depending on the ligand (Glass and Northup, 1999; Bosier et al., 2010). For instance, for the CB_1_ receptor has been reported that, whereas 2-AG and WIN55,212 have little preference for inhibition of cAMP and phosphorylation of ERK1/2, anandamide and CP55940 were biased toward cAMP inhibition (Khajehali et al., 2015). Moreover, in a recent study Dhopeshwarkar and Mackie (2016) demonstrated that CB_2_ receptor ligands display strong and varied functional selectivity at canonical (inhibition of adenylyl cyclase) and non-canonical (arrestin recruitment) pathways. Moreover, the intracellular signaling activated by a receptor depends on the cellular system where it is expressed, which may vary across different neuronal environments. In this context, it has been demonstrated that opioid and cannabinoid receptors function through the same pool of G proteins when they are co-transfected, whereas in cells endogenously expressing these receptors signaling occurs through distinct pools of G proteins (Shapira et al., 2000). Thus, this fact should be taken into consideration when interpreting results acquired in artificially transfected cells vs. native biological systems.

To our knowledge, no study has compared G protein signaling by different cannabinoid drugs in native brain tissue. Thus, in the current study, we performed [^35^S]GTPγS scintillation proximity assay (SPAs) coupled with the use of specific antibodies against different Gα protein subunits to evaluate the functional selectivity of different cannabinoid ligands by activating CB_1_ and/or CB_2_ cannabinoid receptors in mouse brain cortex.

## Materials and Methods

### Animal Procedures

Adult C57BL/6J (WT), CB_1_ knock-out (CB_1_^-/-^) (Marsicano et al., 2002), CB_2_ knock-out (CB_2_^-/-^) (Buckley et al., 2000), and CB_1_/CB_2_ double knock-out (CB_1_^-/-^/CB_2_^-/-^) mice were used in this study. Animals (males, aged 7–8 weeks-old) were housed (6–8 animals per cage) in standard cages under controlled conditions of temperature (23 + 1°C) and photoperiod (light/dark cycle 14 h: 10 h) and free access to standard rodent chow and water.

### Animal Welfare and Ethical Statements

All experimental procedures using mice were performed in accordance with the European Directive for the Protection of Vertebrate Animals used for experimental and Other Scientific Purposes (European Union Directive #86/606/EEC) and approved by the Ethics Committees for Animal Welfare of the University of the Basque Country (UPV/EHU) permit number CEBA1882011 and by the Institutional Review Board (INIA), permit number CEEA2012/021.

### Rationale for Choice of Cannabinoid Ligands

**FIGURE 1:**
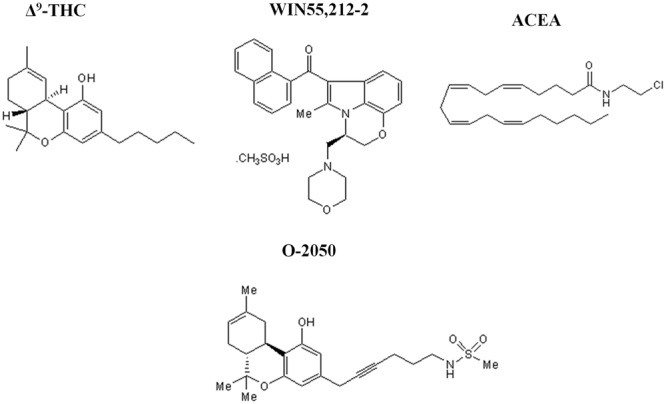
**Chemical structure of the different cannabinoid ligands used.** Representative scheme of the chemical structure of the different cannabinoid ligands used in this study (Δ^9^-tetrahydrocannabinol (Δ^9^-THC), WIN55212-2, arachidonyl-2-chloroethylamide (ACEA) and O-2050).

In the present study, we decided to investigate the effects of three different cannabinoid ligands. Δ^9^-THC was chosen for being the main psychoactive component of marijuana plant and the putative responsible of the development of mental disorders in humans. WIN55212-2, a synthetic cannabinoid structurally different from Δ^9^-THC, is a potent, non-selective CB_1_/CB_2_ receptor agonist that is frequently used in the studies that try to elucidate the effects of *Cannabis* in the brain. Finally, we wanted to study a ligand structurally similar to endogenous cannabinoids, such as the synthetic anandamide analog arachidonyl-2-chloroethylamide (ACEA). O-2050 was chosen as a neutral cannabinoid antagonist. O-2050 has been proved to be a neutral CB_1_ receptor antagonist in several studies (Canals and Milligan, 2008; Hudson et al., 2010; Brents et al., 2011; Wiley et al., 2011), with quite similar affinity for CB_1_ and CB_2_ receptors. Although, there is some data in the literature suggesting its activity as inverse or even partial agonist at CB_1_ receptors in various tissues (Makwana et al., 2010; Wiley et al., 2011) in a previous work of our group, we showed that O-2050 has no effect over [^35^S]GTPγS binding and behaves as an antagonist blocking WIN55212-2-mediated activation (Erdozain et al., 2012) (**Figure 1**).

### Rationale for Choice of G Protein α Subunit Subtypes

**FIGURE 2:**
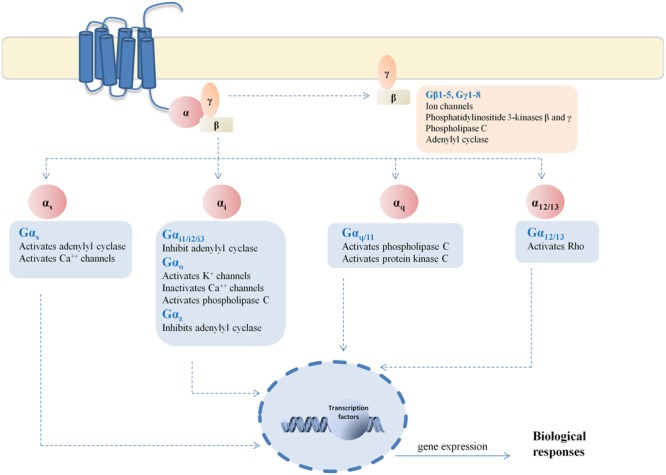
**G protein signaling pathways.** Representative scheme of the signaling pathways linked to each G protein evaluated in this study.

In the present study, we decided to investigate the ability of cannabinoid receptors to activate different G proteins subtype. We chose at least one G protein subtype representative of each main G protein family and mainly focusing in the inhibitory G proteins for being the cannabinoid canonical pathway (**Figure 2**).

### Mouse Brain Cortex Membrane Homogenates Preparation

After sacrifice by cervical dislocation, the brains were rapidly removed, cortices dissected and fresh frozen, and stored immediately at -80°C until use. Preparation of membrane enriched fraction (P2 fraction) was performed as previously described (Gonzalez-Maeso et al., 2000) with minor modifications. Mouse brain cortex samples (approximately 200 mg) from seven mice each time were thawed at 4°C and homogenized with a glass/teflon grinder (IKA labortechnik, Satufen, Germany) (10 strokes at maximum speed) in 30 volumes of homogenization buffer (50 mM Tris-HCl (Invitrogen, Barcelona, Spain), 1 mM EGTA (Sigma-Aldrich, St. Louis, MO, USA), 3 mM MgCl_2_ (Sigma-Aldrich, St. Louis, MO, USA) and 1 mM DTT (Invitrogen, Barcelona, Spain); pH 7.4; supplemented with 250 mM sucrose (Panreac Química S.A.U, Barcelona, Spain). The homogenates were centrifuged at 1,100 × *g* for 10 min at 4°C (Sorvall RC-5C centrifuge, SM-24 rotor; FisherScientific, Madrid, Spain). The pellets (P1 fraction) were discarded and the supernatants were then recentrifuged at 40,000 × *g* for 10 min (4°C). The resultant pellets were resuspended in 20 volumes of fresh cold centrifugation buffer (50 mM Tris-HCl, 1 mM EGTA, 3 mM MgCl_2_ and 1 mM DTT; pH 7.4) with a glass stick and recentrifuged at 40,000 × *g* for 10 min (4°C). The obtained pellets were then resuspended in five volumes of centrifugation buffer. Protein content was determined by the method of Bradford with BSA (Sigma-Aldrich^®^, St. Louis, MO, USA) as standard. Linear regression analysis and extrapolation of the data were carried out with GraphPad Prism 5^®^ software (GraphPad Software, Inc., San Diego, CA, USA). Finally, aliquots of 0.5, 1, and 2 mg were then centrifuged at 21,000 × *g* (Eppendorf 5810R centrifuge; Eppendorf, Madrid, Spain) during 15 min at 4°C. The supernatant layer was carefully discarded and the pellets stored at -80°C until assay.

### Antibody-Capture [^35^S]GTPγS Scintillation Proximity Assay (SPA)

Specific activation of different subtypes of Gα proteins was determined using a homogeneous protocol of [^35^S]GTPγS SPA coupled with the use of specific antibodies essentially as previously described (Erdozain et al., 2012). [^35^S]GTPγS binding was performed in 96-well Isoplates (PerkinElmer Life Sciences, Maanstraat, Germany) and in a final volume of 200 ml containing 1 mM EGTA, 3 mM MgCl_2_, 100 mM NaCl, 0.2 mM DTT, 50 mM Tris-HCl at pH 7.4, 0.4 nM [^35^S]GTPγS, 15 μg of protein per well, and different concentrations of GDP depending on the Gα subunit subtype tested. At the end of the 2 h incubation period (30°C), 20 μl of Igepal 1% + SDS 0.1% were added to each well, and plates were incubated at 22°C for 30 min with gentle agitation. Specific antibody for the Gα subunit of interest was then added to each well before an additional 90 min RT incubation period (the antibodies and dilutions employed are described in **Table 1**). Polyvinyltoluene (PVT) SPA beads coated with protein A (PerkinElmer, S.L., Tres Cantos, Madrid, Spain) were then added (0.75 mg of beads per well), and plates were incubated for 3 h at RT with gentle agitation. Finally, plates were centrifuged (5 min at 1000 × *g*), and bound radioactivity was detected on a MicroBeta TriLux scintillation counter (PerkinElmer S.L., Tres Cantos, Madrid, Spain). In order to test their effect on the [^35^S]GTPγS binding to the different Gα subunit subtypes, a single submaximal concentration of the drugs (10 μM) Δ^9^-THC, WIN55212-2, ACEA and/or O-2050, was used. This submaximal concentration was chosen as previously reported (Erdozain et al., 2012) in our previous experimental assays in which we established the standard conditions for this assays. This concentration is the one which give us binding values around the Emax for any drug and subunit subtype combination studied (Supplementary Figure S1). Non-specific binding was defined as the remaining [^35^S]GTPγS binding in the presence of 10 μM unlabelled GTPγS.

**Table 1:** Antibodies, dilutions, and GDP concentrations employed in the [^35^S]GTPγS scintillation proximity assays.

Target	Description	Commercial firm	Catalog #	[GDP] (μM)	Ab dilution
Gα_i1_	Mouse monoclonal anti-Gα_i1_	Santa Cruz	sc-56536	100	1:20
Gα_i2_	Rabbit polyclonal anti-Gα_i2_	Santa Cruz	sc-7276	50	1:20
Gα_i3_	Rabbit polyclonal anti-Gα_i3_	Santa Cruz	sc-262	200	1:30
Gα_o_	Mouse monoclonal anti-Gα_o_	BIOMOL	SA-280	50	1:75
Gα_z_	Rabbit polyclonal anti-Gα_z_	Santa Cruz	sc-388	100	1:20
Gα_s_	Rabbit polyclonal anti-Gα_s_	Santa Cruz	sc-383	100	1:20
Gα_q/11_	Rabbit polyclonal anti-Gα_q/11_	Santa Cruz	sc-392	50	1:20
Gα_12/13_	Rabbit polyclonal anti-Gα_12/13_	Santa Cruz	sc-28588	100	1:20

### Western Blot

For Western blot experiments, membrane enriched fraction (P2 fraction) pellets from mouse brain tissue (cortex) were resuspended in TBS, reaching a concentration of 4 mg protein/ml. Commercial Laemmli 2x (95%) and β-mercaptoethanol (5%) (Sigma-Aldrich^®^, St. Louis, MO, USA) were added to each sample, reaching a final protein concentration of 2 mg/ml. Finally, all the samples were heated at 95°C for 5 min in a Thermoblock (Biometra, Goettingen, Germany) and kept at -20°C until assay. Electrophoresis was carried out in SDS polyacrylamide gels, composed of 5% stacking (0.5 M Tris-HCl, pH 6.8, 10% SDS) and 12% resolving (1.5 M Tris-HCl, pH 8.8, 10% SDS) gels, using a miniprotean system (Bio-Rad Laboratories). Equal protein loading in the gel was verified by simultaneous immunodetection of β-actin (mouse monoclonal antibody anti-β-actin, Sigma Biosciences, St. Louis, MO, USA) with the different Gα subunit subtypes. Proteins were then transferred to nitrocellulose membranes (1 h, 0.3 A) using an electrophoretic transfer system (Bio-Rad Laboratories). The non-specific binding sites in the membranes were blocked for 1 h at RT in blocking solution (3% non-fat dry milk, pH = 7.4 in PBS). Membranes were incubated overnight at 4°C in incubation buffer (3% non-fat dry milk + 0.1% Tween-20 in PBS) containing the appropriate dilution of the specific primary anti-Gα subunit antibody. Antibody specificity, as previously described in the literature (Gettys et al., 1994; Valdizan et al., 2010), was confirmed in our experimental conditions by Western blot (data not shown). Membranes were washed with PBS and incubated for 1 h at RT and constant agitation with the fluorescent conjugated secondary antibodies (Alexa Fluor^®^ 680 and/or IRDye 800 conjugated antibodies) suitable diluted in incubation buffer. Finally, membranes were rewashed with PBS and immunoreactivity was detected and quantified using the Odyssey Infrared Imaging System (LI-COR Biosciences, Lincoln, NE, USA) and Odyssey Software. Broad-Range pre-stained SDS-PAGE molecular weight standard (Bio-Rad Laboratories, Hercules, CA, USA) was used.

### Data Analysis and Statistical Procedures

Data were analyzed with GraphPad Prism^TM^ 5.01 software. In order to allow better interpretation of the data, specific binding obtained from [^35^S]GTPγS SPAs were transformed to percentage of basal binding (binding values observed in the absence of any exogenous drug) obtained for each Gα protein subunit studied. The statistical comparison of the SPA results was carried out by a two-tailed one sample Student’s *t*-test with a significance level of *p* < 0.05. Immunodensity data obtained from Western blotting assays were transformed to percentage of the control, being the control the mean of immunodensities obtained for WT mice. The statistical comparison of the Western blot results was carried out by a one-way ANOVA test, followed by Dunnet’s *post hoc* test for multiple comparisons, with a significance level of *p* < 0.05. All data are expressed as mean ± SEM values.

### Materials

[^35^S]GTPγS (4625 × 10^10^ Bq/mmol) was purchased from PerkinElmer Life Sciences (Maanstraat, Germany). Tetrahydrocannabinol (Δ^9^-THC) was purchased from THCPharm GmbH (Frankfurt, Germany); WIN55212-2 and GTPγS were purchased from Sigma-Aldrich (St. Louis, MO, USA); Arachidonyl-2-chloroethylamide ACEA and O-2050 were from Tocris Bioscience (Bristol, UK). All other chemical reagents were of analytical quality and were purchased from Merck (Darmstadt, Germany) or Sigma-Aldrich (St. Louis, MO, USA).

## Results

### Effects of Δ^9^-THC, WIN55212-2, and ACEA on G Protein Activation in Mouse Brain Membranes

**FIGURE 3:**
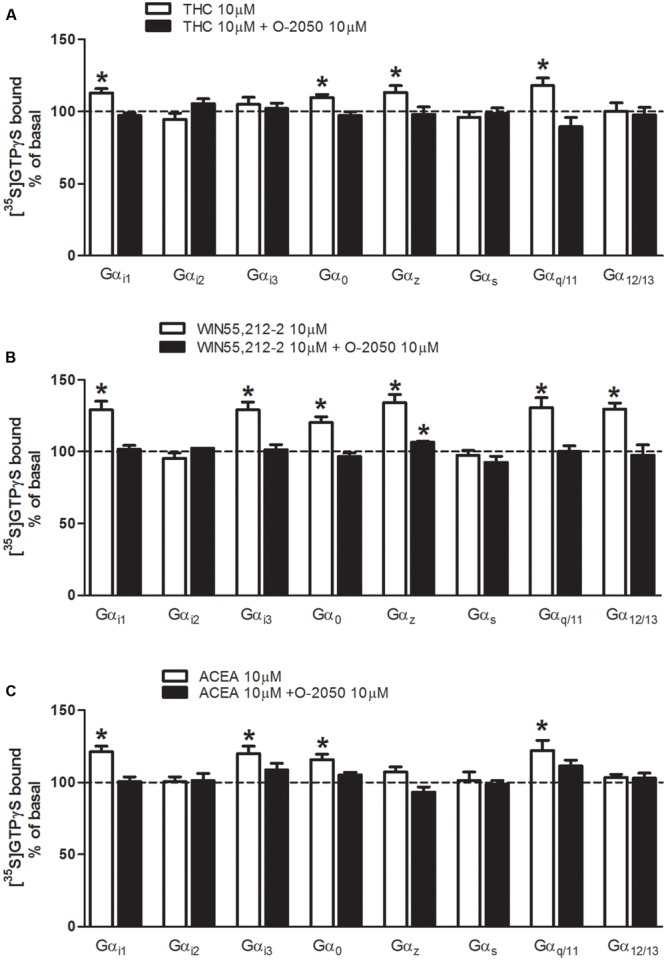
**Effects of THC, WIN55212-2, and ACEA on G protein activation in mouse brain membranes.** [^35^S]GTPγS scintillation proximity assays coupled to immunoprecipitation with specific antibodies against different Gα subunits (Gα_i1_, Gα_i2_, Gα_i3_, Gα_o_, Gα_z_, Gα_s_, Gα_q/11_, and Gα_12/13_) in mouse brain cortical membranes co-incubated with **(A)** THC (10 μM) **(B)** WIN55212-2 (10 μM), or **(C)** ACEA (10 μM) in the presence or absence of the antagonist O-2050 (10 μM). Data are shown as percentage of [^35^S]GTPγS basal binding values obtained for each specific subunit. Bars represent mean ± SEM of four to six different experiments carried out in triplicate. Asterisks highlight those normalized values of stimulation or inhibition of basal binding which are statistically different from 100% (Student’s *t*-test; ^∗^*p* < 0.05).

Cannabinoid receptor ligands were used for the characterization of the functional coupling of cannabinoid receptors to the different G protein α subunit subtypes (Gα_i1_, Gα_i2_, Gα_i3_, Gα_o_, Gα_z_, Gα_s_, Gα_q/11_, and Gα_12/13_) in mouse brain tissue. First, we investigated which Gα subunit subtypes were activated by the natural cannabinoid Δ^9^-THC (10 μM) in mouse brain cortex membrane homogenates (**Figure 3A**). As expected, we found that Δ^9^-THC was able to significantly activate several classical AC inhibitory subunits, as Gα_i1_ (113 ± 3%), Gα_o_ (110 ± 2%), and Gα_z_ (113 ± 5%), while exerted no effect on Gα_i2_ or Gα_i3_. Δ^9^-THC was also able to activate the Gα_q/11_ subunit (118 ± 5%). However, no changes were observed when we studied AC stimulatory subunit Gα_s_ and the RhoA activator Gα_12/13_ subunit. To further test if these effects of Δ^9^-THC were cannabinoid-receptor mediated, the same assays were carried out in the presence of a putative neutral antagonist of the CB_1_ receptor, O-2050. In all cases, the activation of these G protein subunits was blocked when membranes were co-incubated with the cannabinoid antagonist O-2050. Next, we investigated the effects on G protein subunit activation induced by the synthetic cannabinoid agonist WIN55212-2 (**Figure 3B**). We found that WIN55212-2 significantly increased the binding of [^35^S]GTPγS to the all the inhibitory subunits Gα_i1_ (129 ± 6%), Gα_i3_ (129 ± 5%), Gα_o_ (120 ± 4%) and Gα_z_ (134 ± 6%), except Gα_i2_. WIN55212-2 was also able to activate the Gα_q/11_ subunit (131 ± 7%), but not the AC stimulatory subunit Gα_s_. Surprisingly, WIN55212-2 was also able to significantly stimulate the RhoA activator Gα_12/13_ (130 ± 4%). In the same way as previously described for Δ^9^-THC, the activation of these G protein subunits by WIN55212-2 was always blocked by the co-incubation with the cannabinoid antagonist O-2050, except for the case of Gα_z_ (106 ± 1%). Finally, we investigated the effect of the synthetic anandamide analog ACEA on G protein subunit activation in mouse brain tissue (**Figure 3C**). When evaluating the classical AC inhibitory subunits, we found that ACEA stimulated Gα_i1_ (121 ± 4%), Gα_i3_ (120 ± 5%), and Gα_o_ (116 ± 4%). However, as occurred with Δ^9^-THC and WIN55212-2, no stimulation was observed in Gα_i2_, suggesting that none of the cannabinoids evaluated exert their effects through Gα_i2_ signaling. Moreover, ACEA had no effect on Gα_z_. As previously observed for the other two cannabinoid ligands evaluated, ACEA also activated Gα_q/11_ subunit (122 ± 7%) while had no effect on Gα_s_. Thus, it seems that none of these cannabinoid ligands are able to activate this AC stimulatory subunit either. No changes were observed when we studied the effects of ACEA on the RhoA activator Gα_12/13_ subunit. Again, the activation of these G protein subunits was blocked when membranes were co-incubated with the cannabinoid antagonist O-2050.

### Effects of the Cannabinoid Antagonist O-2050 on G Protein Activation in Mouse Brain Membrane Homogenates

**FIGURE 4:**
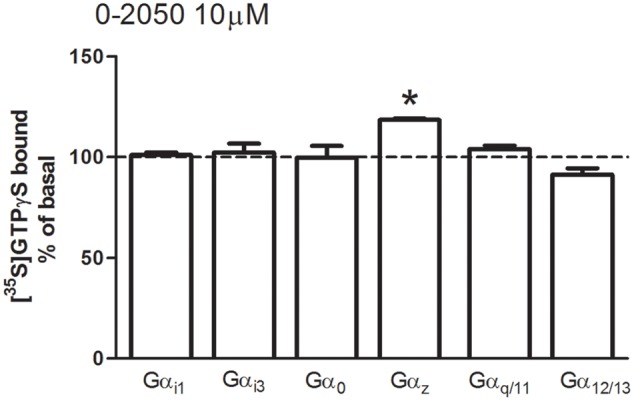
**Effects of the cannabinoid antagonist O-2050 on G protein activation in mouse brain membranes.** [^35^S]GTPγS scintillation proximity assays coupled to immunoprecipitation with anti-Gα_i1_, Gα_i3_, Gα_o_, Gα_z_, Gα_q/11_, and Gα_12/13_ specific antibodies in mouse brain cortical membranes co-incubated with the cannabinoid antagonist O-2050 (10 μM). Data are shown as percentage of [^35^S]GTPγS basal binding values obtained for each specific subunit. Bars represent mean ± SEM of four to six different experiments carried out in triplicate. Asterisks highlight those normalized values of stimulation or inhibition of basal binding which are statistically different from 100% (Student’s *t*-test; ^∗^*p* < 0.05).

O-2050 was initially synthesized and described as a neutral CB_1_ receptor antagonist, however, there are some evidences suggesting that is able to act as an inverse agonist or even as a partial agonist (Wiley et al., 2011). For this reason, and in order to validate O-2050 as a useful pharmacological tool to antagonize the effect mediated by cannabinoid receptors, [^35^S]GTPγS SPAs were performed in mouse cortical membranes in the presence of O-2050 (10 μM) alone. Under these experimental conditions, neither stimulation nor inhibition of [^35^S]GTPγS basal binding values were observed for any of the Gα subunit subtypes studied, with the exception of Gα_z_ (119 ± 1%) (**Figure 4**).

### Effects of Δ^9^-THC, WIN55212-2, and ACEA on G Protein Activation in Cannabinoid Receptors Knockout Mice

**FIGURE 5:**
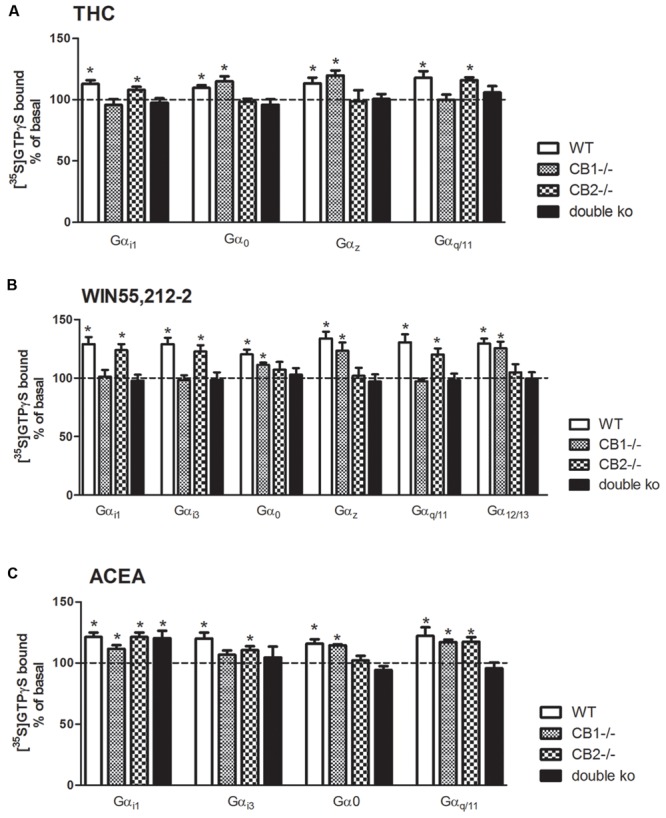
**Effects of THC, WIN55, 212-2, and ACEA on G protein activation in cannabinoid receptors knockout mice.** [^35^S]GTPγS scintillation proximity assays coupled to immunoprecipitation with specific antibodies against different Gα subunits in mouse brain cortical membranes of WT, CB_1_ knockout (CB_1_^-/-^), CB_2_ knockout (CB_2_^-/-^) and double CB_1_ and CB_2_ knockout (CB_1_^-/-^/CB_2_^-/-^/double KO) co-incubated with **(A)** THC (10 μM) **(B)** WIN55212-2 (10 μM), or **(C)** ACEA (10 μM). Data are shown as percentage of [^35^S]GTPγS basal binding values obtained for each specific subunit. Bars represent mean ± SEM of four to six different experiments carried out in triplicate. Asterisks highlight those normalized values of stimulation or inhibition of basal binding which are statistically different from 100% (Student’s *t*-test; ^∗^*p* < 0.05).

To further elucidate the role of each cannabinoid receptor subtype in the agonist-mediated activation of the different Gα subunit subtypes, [^35^S]GTPγS SPA was performed in brain tissue of CB_1_^-/-^, CB_2_^-/-^, and CB_1_^-/-^/CB_2_^-/-^ mice. For that purpose, brain membranes were incubated with the different cannabinoid ligands (THC, WIN55212-2, or ACEA) and with the specific antibodies against that Gα for which an stimulation with these agonists was observed in WT. **Figure 5A** shows the stimulation of the different Gα subunits when brain membranes of the four genotypes were incubated with Δ^9^-THC. The significant stimulation of the inhibitory Gα_i1_ subunit observed in the WT mice was completely absent in the CB_1_^-/-^ and in the CB_1_/CB_2_ double ko mice, but was still present in the CB_2_^-/-^ (108 ± 2%), which suggests that the Δ^9^-THC-mediated stimulation of Gα_i1_ is induced by the activation of CB_1_ receptor. On the other hand, opposite results were obtained for Gα_io_ and Gα_z_, the other two inhibitory subunits that were stimulated by Δ^9^-THC. As previously described, there was a significant Δ^9^-THC-induced stimulation of Gα_o_ and Gα_z_ subunits in the WT. This stimulation was also observed in the CB_1_^-/-^ (115 ± 4% for Gα_o_ and 120 ± 4% for Gα_z_) but not in the CB_2_^-/-^ or the CB_1_^-/-^/CB_2_^-/-^ mice. These data may indicate that, in mouse brain cortical membranes, Δ^9^-THC acts through the CB_2_ receptor to stimulate these inhibitory Gα_o_ and Gα_z_ subunits. Finally, the Δ^9^-THC-induced activation of the Gα_q/11_ subunit observed in the WT mice was not found in the CB_1_^-/-^ and CB_1_^-/-^/CB_2_^-/-^ mice, while remained unchanged in the CB_2_^-/-^ (116 ± 2%). This result indicates that Δ^9^-THC stimulates the Gα_q/11_ subunit acting mainly through the CB_1_ receptor.

**Figure 5B** shows the stimulation of different Gα subunits when brain membranes of the four genotypes were incubated with the synthetic cannabinoid agonist WIN55212-2. The significant stimulation of the inhibitory Gα_i1_ and Gα_i3_ subunits induced by WIN55212-2 in the WT mice was not found in the CB_1_^-/-^ nor in the CB_1_^-/-^/CB_2_^-/-^ mice, but was still present in the CB_2_^-/-^ (124 ± 5% for Gα_i1_ and 123 ± 5% for Gα_i3_). On the contrary, the inhibitory subunits Gα_o_ and Gα_z_, which were significantly stimulated in the WT mice, remained stimulated in the CB_1_^-/-^ (111 ± 2% for Gα_o_ and 123 ± 7% for Gα_z_) but not stimulation was found in the CB_2_^-/-^ nor in the CB_1_^-/-^/CB_2_^-/-^ mice. These results suggest that the inhibitory signaling of WIN55212-2 in the mice brain through Gα_i1_ and Gα_i3_ activation seems to be mediated by the CB_1_ receptor, while the stimulation of Gα_o_ and Gα_z_ would be mediated by the CB_2_ receptor activation. The significant activation of the Gα_q/11_ subunit induced by WIN55212-2 in the WT mice was completely absent in the CB_1_^-/-^ mice, as well as in the CB_1_/CB_2_ double ko mice. On the contrary, a significant stimulation of Gα_q/11_ subunit (120 ± 5%) was observed in the CB_2_^-/-^ membranes, suggesting that this stimulation is mediated by the CB_1_ receptor. Strikingly, the observed stimulation of the RhoA activator subunit Gα_12/13_ by WIN55212-2 in the WT disappeared in the absence of CB_2_ receptor (both in CB_2_^-/-^ and CB_1_^-/-^/CB_2_^-/-^ mice) suggesting an important role of this CB_2_ receptor in the intracellular signaling through Gα_12/13_ in the brain.

Finally, the same experiments were performed incubating the brain membranes with the synthetic anandamide analog ACEA (**Figure 5C**). Surprisingly, the significant stimulation of Gα_i1_ that was observed in the WT mice was still found in all the genotypes evaluated. These results suggest that the inhibitory effect of ACEA mediated by the Gα_i1_ subunit activation may be independent of cannabinoid receptors. On the other hand, the significant stimulation of Gα_i3_ subunit induced by ACEA was not observed in brain membranes of CB_1_^-/-^ mice but was still significant in CB_2_^-/-^ membranes (111 ± 3%). No stimulation was observed in the CB_1_^-/-^/CB_2_^-/-^ mice. Regarding the Gα_o_ subunit, there was a significant stimulation in the absence of CB_1_ receptors (115 ± 1%), while this stimulation was not observed in the brain membranes of CB_2_^-/-^ mice, suggesting the necessary role of this receptor in the activation of Gα_o_ induced by the agonist ACEA. The activation of the Gα_q/11_ subunit was observed in both CB_1_^-/-^ (117 ± 2%) and CB_2_^-/-^ (118 ± 4%) but not in the CB_1_^-/-^/CB_2_^-/-^ mice. Thus, as for Δ^9^-THC and WIN55212-2, the activation of Gα_i3_ and Gα_o_ subunits was mediated by their interaction with CB_1_ and CB_2_ receptors, respectively. However, the stimulation of [^35^S]GTPγS binding to Gα_q/11_ subunit seems to be triggered by the activation of both CB_1_ and CB_2_ cannabinoid receptors.

### Expression of G Protein Subunits in Knockout Mice for Cannabinoid Receptors

In order to determine if a physiological adaptation of knockout mice to the genetic manipulation to inactivate CB_1_ and/or CB_2_ receptors may influence our results by the alteration of the expression level of the different Gα protein subunits, Western blotting assays were carried out in brain cortex membranes of WT, CB_1_^-/-^, CB_2_^-/-^, and CB_1_^-/-^/CB_2_^-/-^ mice.

**FIGURE 6:**
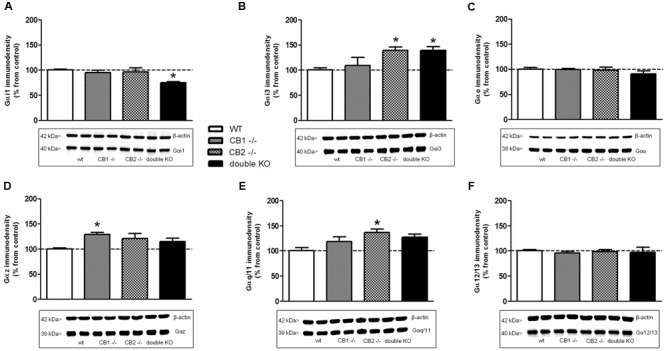
**Expression of G protein subunits in cannabinoid receptors knockout mice.** Immunoreactive signal and representative images obtained by Western blotting with specific antibodies against different Gα subunits: **(A)** Gα_i1_, **(B)** Gα_i3_, **(C)** Gα_o_, **(D)** Gα_z_, **(E)** Gα_q/11_, and **(F)** Gα_12/13_ in mouse brain cortical membranes of WT, CB_1_ knockout (CB_1_^-/-^), CB_2_ knockout (CB_2_^-/-^) and double CB_1_ and CB_2_ knockout (CB_1_^-/-^/CB_2_^-/-^/double KO). Immunoreactivity for β-actin was simultaneously detected on every gel and used as loading control. Normalized values (percentage over controls) of Gα subunits are shown as mean ± SEM of two different experiments carried out in duplicate. Asterisks highlight those values which are statistically different from WT (One-way ANOVA followed by Dunnet’s *post hoc* test; ^∗^*p* < 0.05).

In the case of Gα_i1_ (**Figure 6A**), no changes were observed for CB_1_^-/-^ and CB_2_^-/-^, but a significant reduction (33 ± 10% from WT) of immunodensity was detected in CB_1_^-/-^/CB_2_^-/-^ mice brain membrane homogenates. No significant differences were found in the expression of Gα_o_ (**Figure 6C**) or Gα_12/13_ (**Figure 6F**) subunits between the WT, CB_1_^-/-^, CB_2_^-/-^, and CB_1_^-/-^/CB_2_^-/-^ mice brain membranes. However, an increase in the expression of Gα_i3_ (**Figure 6B**) in both CB_2_^-/-^ (139 ± 7%) and CB_1_^-/-^/CB_2_^-/-^ mice (140 ± 7%) was found when compared to the WT and CB_1_^-/-^ animals. On the contrary, Gα_z_ immunodensity was significantly increased in the CB_1_^-/-^ mice (129 ± 4%) while no changes were found in the rest of the genotypes when comparing to the WT (**Figure 6D**). Finally, the expression of Gα_q/11_ (**Figure 6E**) was significantly increased in brain membranes of CB_2_^-/-^ mice (137 ± 7%) but not in WT, CB_1_^-/-^ and CB_1_^-/-^/CB_2_^-/-^ mice.

## Discussion

During the last years, a considerable effort has been made to study the effects of cannabinoids in the brain trying to elucidate the mechanisms by which these compounds may facilitate mental disorders, such as addiction, cognitive deficits, anxiety or psychosis. In this context, these studies have been performed with different cannabinoid ligands (natural or synthetics), in cells or in native tissue and/or in different animal species (mouse, rat, human…).

There is wide evidence supporting the idea that for most of GPCRs, distinct drugs are able to regulate different signaling pathways by the selective activation of different intracellular effectors. The pharmacological relevance of this fact is that the biological responses not only depend on targeting a specific GPCR but also on the particular pathway that this receptor activates. Different studies have focused on the evaluation of the functional selectivity of cannabinoid receptors, but most of them have been performed in transfected cells expressing the CB_1_ receptor (Glass and Northup, 1999; Bosier et al., 2010). Moreover, much of these studies explore the signaling pathways activated by different agonists by the evaluation of cAMP production or the phoshorylation of intracellular mediators such as ERK or AKT, with no data about the Gα subtype responsible of these downstream effects. These changes on cAMP concentration or ERK/AKT phosphorylation could be the consequence of the activation of different Gα subtypes, Gβγ dimmers, etc. On the other hand, and interestingly, opposite behavioral effects have been observed after the administration of Δ^9^-THC or synthetic cannabinoid ligands (Fattore et al., 2003; Panagis et al., 2014; Rubino and Parolaro, 2016). For example, when evaluating the cannabinoid effects on brain-stimulation reward, Fattore et al. (2003) showed that the potent non-selective CB_1_/CB_2_ receptor agonists WIN55,212-2 and CP 55,940, but not Δ^9^-THC, effectively restored heroin-seeking behavior. In addition, it has been suggested that the signaling of CB_1_ receptors may differ between humans and rodents (Straiker et al., 2012).

All these frequently contradictory data highlight the relevance of studying, simultaneously, the effects of different cannabinoid ligands in the same tissue and under the same experimental conditions.

For that reason the goal of the present study was to compare the pattern of G protein subunit stimulation triggered by three structurally different cannabinoids, Δ^9^-THC, WIN55212-2 and ACEA in mouse brain cortex. To our knowledge, this is the first study evaluating the cannabinoid-induced stimulation of the different Gα subunits in mouse brain tissue.

WIN55212-2, a synthetic cannabinoid structurally different from Δ^9^-THC, is a potent, non-selective CB_1_/CB_2_ receptor agonist that has been used in many studies of cannabinoid receptor function (Pertwee et al., 2010). The synthetic anandamide analog ACEA is a highly selective agonist for the CB_1_ receptor with a low affinity for CB_2_ receptors (Hillard et al., 1999).

This study demonstrates that each ligand displays functional selectivity acting as biased agonist for a subset of different G protein subunits. It represents the first characterization of the activation of individual Gα subunits by endogenous cannabinoid receptors in brain cortex. Firstly, we demonstrated that phytocannabinoid Δ^9^-THC differs from the synthetic agonists WIN55212-2 and ACEA in its ability to stimulate Gα_i/o_ protein subunits in brain cortex.

The Gα_i_ subfamily members Gα_i1_, Gα_i2_, and Gα_i3_ were originally identified by their ability to inhibit AC activity (Plummer et al., 2012; Busnelli et al., 2013; Minetti et al., 2014). Our results show that Δ^9^-THC, WIN55212-2, and ACEA significantly stimulate Gα_i1_ subunit. Moreover, data from knockout mice suggest that this effect may be CB_1_-mediated in the case of Δ^9^-THC and WIN55 212-2. However, the Gα_i1_ stimulation is still significant in membranes of all genotypes incubated with ACEA, suggesting that this is a non-CB_1_ non-CB_2_ dependant effect and supporting putative actions of ACEA over other receptors (Pertwee et al., 2010). Gα_i3_ subunit was also stimulated in the presence of WIN55212-2 and ACEA, but not of Δ^9^-THC. This stimulation seems to be mediated by CB_1_ receptors as is blocked in the presence of O-2050 and absent in CB_1_^-/-^ or CB_1_^-/-^/CB_2_^-/-^ mice. In the case of Gα_i2_, it has been previously described that WIN55212-2 is able to activate this subunit in rat (Prather et al., 2000) and in human brain cortical membranes (Erdozain et al., 2012). However, none of the agonists in the present study stimulated the Gα_i2_ subunit. This discrepancy may be due to inter-specie and/or regional differences, suggesting that WIN55212-2 may signal through different G protein pools in human and mouse brain cortex.

These three Gα_i_ subunits form the Gα_i/o_ subfamily with the neuronal α-subunit Gα_o_, which corresponds to the most abundant Gα protein in brain (Sternweis and Robishaw, 1984). In our experimental approach, and in accordance with other studies (Glass and Northup, 1999; Presley et al., 2016), Δ^9^-THC, WIN55212-2 and ACEA significantly stimulated Gα_o_. Results obtained in knockout animals show that the stimulation of Gα_o_ in mouse cortex is mediated, at least in part, by CB_2_ receptors, suggesting a necessary role of this receptor in the cannabinoid-induced activation of Gα_o_.

The Gα_z_ subtype is the most divergent member of the inhibitory subfamily and is distributed primarily in neuronal and neuroendocrine cells (Hinton et al., 1990). While Δ^9^-THC and WIN55212-2, similarly, stimulated Gα_z_, no stimulation of this subunit was observed when membranes were incubated with ACEA, suggesting that ACEA may not signal through this subunit. Additionally, results obtained with knockout mice suggest that the stimulation of Gα_z_ by Δ^9^-THC and WIN55212-2 may be induced by a CB_2_-mediated mechanism.

Unlike Gα_z_, the Gα_s_ family is ubiquitously expressed and couples receptors to AC in a stimulatory fashion (Milligan and Kostenis, 2006). Under the present assay conditions, nor Δ^9^-THC, WIN55212-2 or ACEA were able to activate this stimulatory subunit. Thus, there is no evidence of Gα_s_ coupling of cannabinoid receptors in the presence of any of these drugs in brain tissue. There are contradictory results about the ability of cannabinoid drugs to activate Gα_s_ proteins. In this way, there are data from both CHO cell lines (Rinaldi-Carmona et al., 1996; Bonhaus et al., 1998) and HEK cells (Presley et al., 2016) expressing CB_1_ receptor, showing the absence of effect as well as a modest but significant coupling of CB_1_ to Gα_s_ triggered by different cannabinoids. It has been proposed that ACEA may elevate cAMP through a non-CB_1_ mechanism, since there is an increase in cAMP in both cells transfected and non-transfected with CB_1_ and pretreated with pertussis toxin (Presley et al., 2016). It is important to point out that all these studies have been performed in cell lines. Moreover, they use the accumulation of cAMP in the presence of the Gα_i/o_ inhibitor pertussis toxin as an indirect evaluation of potential coupling of CB_1_ receptors to Gα_s_. This increase in cAMP production can be mediated by a mechanism different from Gα_s_ activation, as they did not explore directly the activation of this subunit. Therefore, the possible increase in cAMP induced by other actors different from Gα_s_ subunits could not be discarded.

The Gα_q/11_ proteins, widely expressed through the CNS, mediate PLC activation, leading to the activation of downstream calcium signaling pathways including PKC and MAPKs activation (Sanchez-Fernandez et al., 2014). In this study, a significant stimulation of Gα_q/11_ was observed in the presence of the three cannabinoids evaluated. It has been previously reported that WIN55212-2 induces the coupling of CB_1_ to Gα_q/11_ in different cellular types (Lauckner et al., 2005; McIntosh et al., 2007). Our results show that not only WIN55212-2 but also Δ^9^-THC and ACEA can activate Gα_q/11_ subunit in mouse brain. Moreover, the activation of this subunit induced by Δ^9^-THC and WIN55212-2 seem to be mediated by the CB_1_ receptor, as demonstrate the data obtained with knockout animals. In the case of ACEA, our data suggest that ACEA modulate Gα_q/11_ through both CB_1_ and CB_2_ cannabinoid receptors.

The Gα_12/13_ proteins regulate important signaling events by the activation of the small GTPase protein RhoA, involved in the regulation of the actin cytoskeleton and cell motility (Kozasa et al., 2011; Yu and Brown, 2015). Under our experimental conditions, a significant stimulation of Gα_12/13_ subunit was observed when membranes were incubated with WIN55212-2 but not with Δ^9^-THC or ACEA. To our knowledge, this is the first study reporting that WIN55212-2 signals through Gα_12/13_ in brain cortex. These data are concordant with other studies suggesting that cannabinoids induce the stimulation of this RhoA-activator (Dalton et al., 2013; Roland et al., 2014). Moreover, our results from knockout mice show that the WIN55212-2-induced signaling through Gα_12/13_ in the brain seems to be mediated, mainly, by the CB_2_ receptor.

Although O-2050 had been described as a CB_1_ antagonist, it displays a complex pharmacological profile. In this context, its good affinity for CB_2_ receptors complicates its use as a tool to evaluate the unique contribution of CB_1_ receptor (Wiley et al., 2011). We observed that, when alone, O-2050 activated the Gα_z_ subunit. Therefore, in co-incubations, O-2050 behaved always as an antagonist of the effects of Δ^9^-THC, WIN55212-2 and ACEA over all the studied Gα subunit subtypes, except for the Gα_z_ subunit. When WIN55212-2 and O-2050 were co-incubated, the stimulation of Gα_z_ was lower but still significant. In this way, the blockade exerted by O-2050 pharmacologically confirmed the involvement of cannabinoid receptors in the observed stimulations.

Studies in knockout mice provide very valuable data in basic research but in addition to the absence of the targeted protein, we cannot discard the appearance of putative neurodevelopmental compensatory mechanisms. In this work, we have used CB_1_ and/or CB_2_ receptor knockout mice to elucidate the role of each receptor in the observed effects of different ligands on the stimulation of Gα subunits. Moreover, Western blotting assays were carried out in order to unmask the role of a possible adaptation of cannabinoid receptors knockout mice affecting the expression level of the different Gα subtypes on the different genotypes. The observed stimulations in knockout mice may not be influenced by putative neurodevelopmental compensatory mechanisms involving G proteins density. In this way, although expression of some Gα subunits in knockout mice is different from the WT, these changes do not explain the absence of stimulation in CB_1_ or CB_2_ knockout mice. The convergence of our pharmacological and genetic data demonstrate that the results obtained herein with the cannabinoid receptors knockout mice are likely due to the absence of the CB_1_ and/or CB_2_ receptors and not to non-specific changes due to neurodevelopmental adaptations.

## Conclusion

Our results demonstrate that, in mouse brain native tissue and under our experimental conditions different exogenous cannabinoids are able to selectively activate different inhibitory and non-inhibitory Gα protein subtypes, through the activation of CB_1_ and/or CB_2_ receptors. However, it is important to be aware of potential limitations. It has been suggested that the signaling of CB_1_ receptors is significantly diminished in humans compared to that of rodents, a finding that may have implications for the use of rodent models for studies of CB_1_ receptor function related to human disease and therapy (Straiker et al., 2012).

Results of the present study may help to dissect the specific signaling pathways involved in the different pharmacological actions of cannabinoids. Moreover, the knowledge of the specific molecular target responsible of these different physiological effects will help in the design of new biased cannabinoid drugs with more specific therapeutic effect and a reduced range of adverse effects.

## Author Contributions

RD-A, II-L, and AL-C performed the experiments, LC, EA, AG-A, JM, and LU designed the study, JM, LC, RD-A, AG-A, and LU analyzed and interpreted the results, RD-A and LU drafted the manuscript. All the contributors revised critically and gave their approval to the final version of the manuscript.

## Conflict of Interest Statement

The authors declare that the research was conducted in the absence of any commercial or financial relationships that could be construed as a potential conflict of interest.

## Abbreviations

2-AG: 2- arachidonoylglycerol
ACEA: arachidonyl-2-chloroethylamide
CB_1_^-/-^: CB_1_ knock-out mice
CB_2_^-/-^: CB_2_ knock-out mice
CB_1_^-/-^/CB_2_^-/-^: CB_1_ and CB_2_ double knock-out mice
P1: pellet fraction
P2: membrane enriched fraction
PVT: polyvinyltoluene
RT: room temperature
SDS: sodium dodecyl sulfate
SPA: scintillation proximity assay
TBS: tris-buffered saline
WT: wild type
Δ^9^-THC: Δ^9^-tetrahydrocannabinol
