# Supplementary figures and images for: Biased Agonism of Three Different Cannabinoid Receptor Agonists in Mouse Brain Cortex
Source: Front Pharmacol. 2016 Nov 4;7:415. doi: 10.3389/fphar.2016.00415 (PMC5095132; doi:10.3389/fphar.2016.00415)

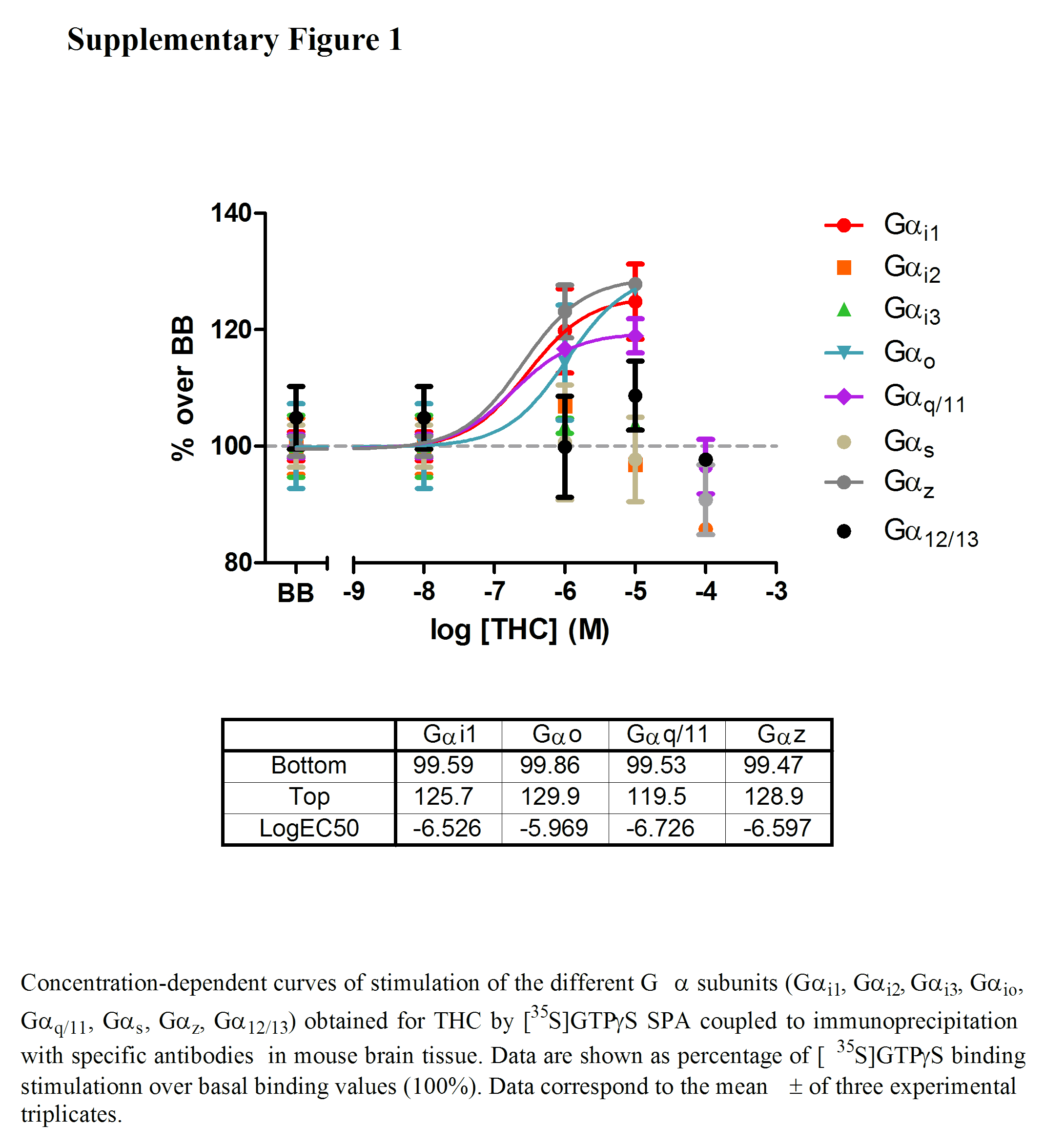

Supplement: Supplementary file 1 [file Image_1.TIF]
